# Health-related quality of life in pediatric and adolescent patients with transfusion-dependent ß-thalassemia in upper Egypt (single center study)

**DOI:** 10.1186/s12955-018-0893-z

**Published:** 2018-04-10

**Authors:** Gehan L Abdel Hakeem, Suzan O Mousa, Asmaa N Moustafa, Mohamed H Mahgoob, Ebtesam E Hassan

**Affiliations:** 1 Pediatric Department, Faculty of Medicine, Minia University, El Minya, 61511 Egypt; 2 Public health, Faculty of Medicine, Minia University, Minia, 61511 Egypt

## Abstract

**Background:**

Thalassemia is a major health problem that disturbs the lifestyle of the affected patient. The aim of this work is to detect the impact of thalassemia on the quality of life regarding physical, social, emotional, psychological scored assessment.

**Methods:**

A case-control survey was conducted in Minia University children’s hospital on 64 patients recruiting pediatric hematology outpatient clinic from July 2014 to February 2017. PedsQL™ 4.0 Generic Core Scale (Arabic version) was used to assess HRQOL in 64 thalassemia patients between 8 and 18 years of ages. Other related clinical data of the involved patients were collected from the pediatric hematology records.

**Results:**

Mean physical, emotional, social, school performance, psychological and total scores (− 36.9 ± 20.9, 49.4 ± 17, 47.2 ± 21.3, 38.5 ± 15.5, 45.3 ± 13.8, 47.9 ± 38.8 respectively) were significantly decreased compared with control (*p* = 0.001 for all). The younger age group had better scores regarding social, emotional, psychological and total scores compared to older ones (*p* = 0.01, 0.03, 0.01 and 0.009 respectively). Older age of starting transfusion was statistically significant protecting factor from poor physical QOL in thalassemia patients (OR = 0.96, *p* = 0.03). The presence of hepatomegaly was a statistically significant predictor for poor physical QOL (OR = 8.5, *p* = 0.02). Household income was the statistically significant predictor for poor emotional QOL (OR = 5.03, *p* = 0.04). High serum ferritin was the statistically significant predictor for poor social QOL (OR = 1.1, CI 95%=, *p* = 0.04). Regarding poor psychological QOL (OR = 0.94, *p* = 0.01) and total QOL (OR = 0.94, *p* = 0.01) scores, older age of starting transfusion was the statistically significant protecting factor.

**Conclusion:**

Scheduled programs giving psychosocial help and a network connecting between the patients, school officials, thalassemia caregivers and the physician is required especially in developing countries where the health services are not integrated with social organizations. Special school services for thalassemia patients are required to deal with the repeated absence and anemia induced low mental performance of thalassemia children.

## Background

Beta-thalassemia is one of the most common autosomal recessive disorders worldwide with high prevalence in the Mediterranean, Middle-East and Central Asia [1]. Beta-thalassemia is caused by the reduced or absent beta globin chain synthesis of hemoglobin (Hb) tetramer, which is made up of two alpha globin and two beta globin chains (alpha_2_beta_2_). The clinical severity of beta-thalassemia is related to the imbalance between the alpha globin and non-alpha globin chains [2–4].

Individuals with Beta-thalassemia major usually present with failure to thrive and progressive pallor requiring regular blood transfusions to survive, abdominal enlargement, caused by splenomegaly and the risk of developing iron overload related complications. Complications of iron overload include growth retardation and failure of sexual maturation. Late complications are cardiac (dilated myocardiopathy and pericarditis), hepatic (chronic hepatitis, fibrosis, and cirrhosis), endocrinal (resulting in diabetes mellitus and parathyroid, thyroid, pituitary, and, less commonly, adrenal glands insufficiencies) and hypersplenism [5–7].

In Egypt, β-Thalassemia is the most frequent hemoglobinopathy. The carrier rate of this disease varies between 5.3-9% and the gene frequency is 0.03%. It was estimated that 1000/ 1.5 million per year live birth born with thalassemia disease [8]. Children with transfusion-dependent thalassemia typically should undergo blood transfusions once or twice a month depending on the severity of the illness. This may force them to spend the entire transfusion day at the hospital with subsequent disruption in education and social activities. Iron overload with the undesirable bronzed color may compromise their body image. With regular deferoxamine Injection therapy for iron chelation, they become increasingly dependent upon others [9]. Adding to these, the complications of iron overload which may become evident when chelation is not feasible or due to lack of patient compliance.

Overall patient’s life, such as education, free-time, physical activities, skills, capabilities, and family adjustment is affected. The effects of which often result in psychological, emotional and social compromise [10].

Health-Related Quality of Life (HRQoL) measurement is a multidimensional concept that focuses on the impact of the disease and its treatment on the wellbeing of an individual. The measures are seen as ways of capturing patients’ perspectives of their disease and treatment, their perceived need for health care and their preferences for treatment and disease outcomes [11].

Varni et al., 2003 created the multidimensional PedsQL 4.0 questionnaire to measure the essential core domains for pediatric HRQoL: Physical functioning, Emotional functioning, and social functioning, as delineated by the World Health Organization (WHO), as well as School functioning [12, 13].

Some recent searches studied the HRQoL of B-thalassemia children and adults and their parents. They found that treatment and cultural differences did not have a major effect on the Quality of life in Cypriot thalassemia patients [14]. Another study on children compared the quality of life in patients with thalassemia intermedia to thalassemia major and discovered that transfusion-independent thalassemia patients also suffer impairments in different quality of life aspects [15]. They concluded that all patients with thalassemia should undergo QOL assessment so that interventions focused on the affected domain can be implemented.

In this study, we aimed to evaluate the quality of life in children and adolescents with thalassemia in El Minya governorate, at Upper Egypt, and to investigate which factors can lead to the establishment of good supportive clinical programs that will improve quality of life in these patients especially that stem cell transplantation is not an option for most of them.

## Methods

This case-control study was done at the pediatric hematology unit in Minia University children’s hospital from July 2014 to February 2017. The enrolled patients were known recorded thalassemia patients that had regular visits to pediatric hematology unit either for clinical and laboratory follow-up or regularly scheduled blood transfusion. The patients were asked to answer the questionnaire during their regular planned visits to the hematology clinic. One hundred eligible beta-thalassemia patients were invited to participate in this study. Only 64 of them agreed to participate.

### Eligibility

Beta thalassemia children fulfilling the following criteria during the study period were included: having an age range between 8 and 18 years, receiving a blood transfusion on a monthly or near-monthly basis.

### Exclusion criteria

We excluded from the study: (1) Beta-thalassemia patients suffering from any acute illness that made it difficult to answer the questionnaire such as acute heart failure, acute, thalassemia patients with any associated chronic disease not related to thalassemia or its complications.

### Control group

Sixty-four healthy age and sex matched students selected during school days were invited to participate in this study as a control group. They were apparently clinically healthy confirmed by a complete blood count.

For all enrolled patients, fully recorded data about their disease history including disease onset, the age of first blood transfusion, the amount of blood transfusion calculated per weight yearly, type, doses and duration of chelation therapy. Initial investigations including complete blood count (CBC), serial serum ferritin measures and hepatitis B and C serology were also recorded. All patients were treated according to the international thalassemia federation guidelines.The cut-off value for serum ferritin to start iron chelation therapy was > 1000 ng/ml [16].

Full physical examination to the enrolled patients including anthropometric measures (weight, height, and head circumference) plotted to the child disease center (CDC) growth charts to be expressed as percentile numbers [17] in addition to complete chest, heart, and abdominal examinations.

The study was explained in detail to the participant children and written consents were taken from their parents or legal guardians after assuring confidentiality of the data. The study was conducted according to the principles of Helsinki and the protocol of the study was approved by the Institutional Review Board and Medical Ethics Committee of Minia University hospital.

### Research instruments

HRQoL was assessed with the PedsQoL 4.0 Generic Core Scales. This instrument has 23-items that are designed to measure the core dimensions of health as delineated by WHO. The PedsQoL 4.0 encompasses the essential core domains for pediatric HRQoL measurement: 1) Physical functioning (8 items), 2) Emotional functioning (5items), 3) Social functioning (5 items) and 4) School functioning (5 items). It consists of developmentally appropriate forms for ages 2–4, 5–7, and 8–12 and 13–18 years. The reliability, validity, responsiveness, and practicality of the PedsQoL Generic Core Scales have been assessed in both physically healthy pediatric populations and in pediatric acute and chronic health conditions. The internal consistency reliability of the PedsQoL 4.0 Generic Core Scale approached 0.90 for self-report [11, 12, 18]. The scores for each dimension were in alignment with the recommended approach and were calculated as follows: the mean score is represented by the sum of the items over the number of items answered; missing values (which were minimal in our study) were replaced by the mean score of the remaining items, Raw scores are transformed into standardized scores on a scale from 0 to 100 with higher scores representing higher functioning levels. The validity of the PedsQoL Generic Core Scales has been demonstrated through known group comparisons and correlations with other measures of disease burden.

User agreement was signed with MAPI Research Institute, Lyon, France prior to using the questionnaires. PedsQL 4.0 Generic Core Scales has also been translated into many languages and we used Arabic for Egypt version provided by MAPI Research Institute [18].

### Data analysis

Data were computed and analyzed by and SPSS (Statistical Package for the Social Sciences) program version 19.0. General characteristics of the patients were presented in terms of percentage, mean, and standard deviation and median for data not normally distributed. For QoL, both total HRQoL score and physical, emotional, social, school achievement and psychological scores were presented in terms of mean and standard deviation. Pearson’s correlation, chi-square, ANOVA, and t-test were used to examine the relationship between HRQoL and each demographic/clinical data. Non-parametric tests were used if data were not normally distributed. Factors influencing the quality of life of children with thalassemia were later examined by multiple regression analysis.

## Results

**Fig. 1:**
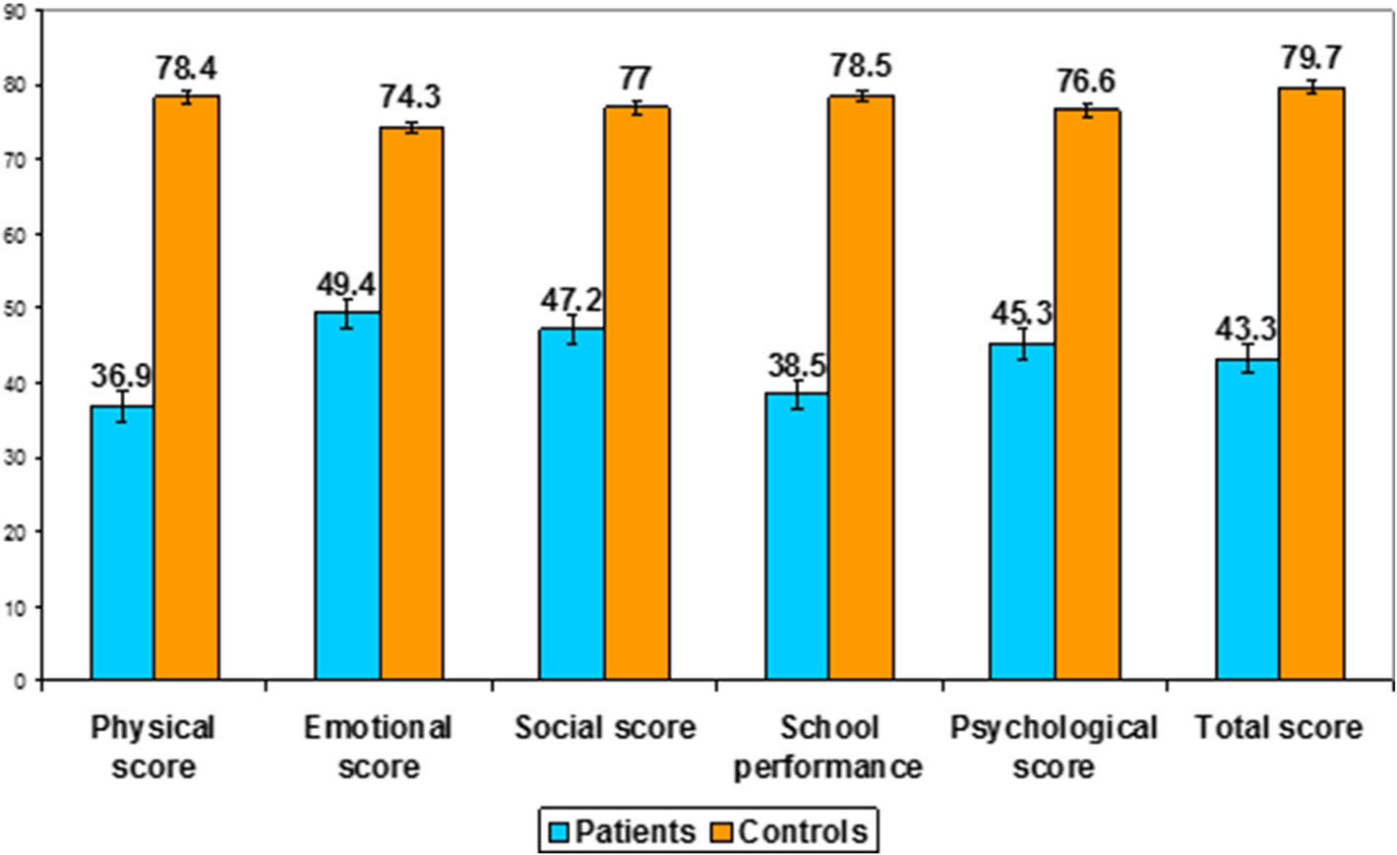
QOL scores in thalassemia children and control: (n = 64 cases and 64 control); *p* < 0.001 in all scores

Sixty-four known thalassemia patients were included. Forty-three (67.2%) of them were males while 21(32.8%) were females. Their age range was 8-18 years with a mean of 12.1 ± 3.2. Another 64 apparently healthy age and sex matched children were enrolled as controls. Their age range was 8-18 years with a mean of 11.1 ± 2.8. Significant differences between beta thalassemia patients and the control group regarding mean weight centile (25.9 ± 18.5 for thalassemia patients and 63 + 19.3 for controls) and height centile (5.7 ± 10.5 for thalassemia patients and 56.1 ± 22.8 for controls) (*p* values = 0.001 for both) (Table 1). Mean physical score was 36.9 ± 20.9 for patients and 78.4 ± 5.5 for controls and was significantly different (*p* = 0.001). For the emotional score, it was 49.4 ± 17 for patients and was 74.3 ± 5.5 for controls with a significant difference (*p* = 0.001). Regarding mean social score, it was 47.2 ± 21.3 in patients while was 77 ± 13.9 in the control group with a significant difference (*p* = 0.001). Mean school achievement score was 38.5 ± 15.5 in patients while was 78.5 ± 6.4 in the control group with a significant difference (*p* = 0.001). The mean psychological score was 45.3 ± 13.8 in patients while was 76.6 ± 3.2 in the control group with a significant difference (p = 0.001). The total QoL score mean was 47.9 ± 38.8 in thalassemia patients while was 79.7 ± 5.9 in control group with a significant difference (*p* = 0.001) (Fig. 1). There were significant differences between children (8-12 years) and teens (13-18 years) regarding emotional, social and psychological scores (*p* = 0.03, 0.013 and 0.04 respectively). Comparing the physical and total QoL scores to the demographic and laboratory data, no significant differences were found (Table 2).

**Table 1:** Demographic, clinical and laboratory data of studied children

Parameters		Patients	Controls	*p*-value
		*N* = 64	*N* = 64	
Age	Range	8-18	8-18	0.16
	Mean ± SD	12.1 ± 3.2	11.1 ± 2.8	
	median	10	12	
Sex	Male	43 (67.2%)	53 (82.8%)	0.15
	female	21 (32.8%)	11 (17.2%)	
Weight centile	Range	5-90	25-95	0.001*
	Mean ± SD	25.9 ± 18.5	63 ± 19.3	
	Median	30	55	
Height centile	Range	5-90	10-95	0.001*
	Mean ± SD	15.7 ± 10.5	56.1 ± 22.8	
	Median	20	59	
Head circumference centile	Range	18-90	15-95	0.22
	Mean ± SD	68.2 ± 19	63.2 ± 16.1	
	Median	75	79	
Educational status:	-Uneducated	20 (31.2%)	0(0%)	0.005*
	-Primary school	31 (48.4%)	50 (78.1%)	
	-Secondary school	13 (20.4%)	14 (21.9%)	
Household income	< 500 EGP	15 (23.4%)	20 (30%)	0.02*
	500-1000	34 (53.2%)	24 (40%)	
	> 1000	15 (23.4%)	20 (30%)	
Age of start transfusion (month)	Range	6-72	–	
	Mean ± SD	15.8 ± 11.8		
	Median	10.5		
Age of start chelation	Range	2-12		
	Mean ± SD	4.1 ± 2.6		
	Median	4		
Type of chelation used	No chelation	7 (10.9%)		
	Deferoxamine	20 (31.3%)		
	Deferoperone	13 (20.3%)		
	Desferosirax	24 (37.5%)		
Liver enlargement	Hepatomegaly	49 (76.6%)		
	No hepatomegaly	15 (23.4%)		
Splenectomized	No	34 (53.1%)		
	Yes	30 (46.9%)		
Hepatitis C infection	Positive	22 (34.4%)		
	Negative	42 (65.6%)		
Hepatitis B infection	Positive	0 (0%)		
	Negative	64 (100%)		
Serum ferritin (ng/ml)	Mean ± SD	975 ± 22.1	43 ± 3.7	0.001*

*= Significant (*p* < 0.05)

**Table 2:** Comparison between different HQOL scores regarding demographic and laboratory data

Parameter		Physical score		Emotional score		Social score		School Achievement score		Psychological score		Total score	
		Mean ± SD	*p* value	Mean	*p* value	Mean	*p* value	Mean	*p* value	Mean	*p* value	Mean	*p* value
Age	8-12	40.3 ± 22.6		53.1 ± 16.8		52.4 ± 21.7		40.8 ± 16.2		47.9 ± 15.2		46.8 ± 15.9	
	13-18	31.6 ± 17.1	0.12	43.5 ± 16.2	0.03*	39.04 ± 18.1	0.013*	34.9 ± 13.9	0.14	41.1 ± 10.2	0.04*	37.7 ± 10.7	0.09
Sex	Male	39.5 ± 22.3		49.1 ± 16.8		48.1 ± 20.5		40.2 ± 14.6		45.6 ± 14.1		44.3 ± 14.6	
	female	31.6 ± 16.9	0.16	49.9 ± 17.7	0.85	45.2 ± 23.2	0.62	34.9 ± 19.02	0.2	44.5 ± 13.4	0.78	41.2 ± 15.1	0.29
Household income	< 500	54.1 ± 16.1		56 ± 19.1		61.6 ± 13.1		47.5 ± 14.7		52.4 ± 10.1		54.3 ± 9.1	
	500-1000	33.2 ± 21.8	0.45	46.6 ± 18.1	0.04*	44.1 ± 23.5	0.9	35.8 ± 15.3	0.78	43.8 ± 15.4	0.27	40.6 ± 16.1	0.39
	> 1000	28.3 ± 13.5		48.7 ± 10.5		39.9 ± 16.8		35.6 ± 14.2		41.4 ± 11.3		38.3 ± 11.1	
Type of chelation	Non	32.1 ± 19.6		41.8 ± 13.6		43.8 ± 19.7	0.54	38.1 ± 16		40 ± 9.4		38.9 ± 10.4	
	Oral	49.6 ± 24.2	0.5	55.3 ± 18.8	0.82	58 ± 25.1		41.9 ± 16.6	0.27	54.9 ± 10.8	0.42	51.8 ± 16.4	0.44
	Sc	39.7 ± 18.8		55.5 ± 17.6		51.5 ± 18.7		44.3 ± 12.6		48.9 ± 11.8		48 ± 11.4	
Splenectomy	No	37.3 ± 23.9	0.54	52.1 ± 23.9	0.46	48.9 ± 24.1	0.63	40.7 ± 16.3	0.74	44.1 ± 9.8	0.08	45 ± 17.5	0.3
	Yes	36.5 ± 17.3		46.3 ± 14.9		45.2 ± 17.9		36.1 ± 14.5		44.9 ± 17.5		41.4 ± 10.9	
Educational level	Primary school	34.9 ± 19.8	0.81	51.2 ± 16.4	0.46	43.7 ± 15.3	0.72	35.7 ± 14.4	0.21	43.6 ± 13.1	0.29	41.3 ± 13.1	0.27
	Secondary school	39.7 ± 21.9		52 ± 17.33		50.6 ± 23.6		40.04 ± 16.6		47.1 ± 15.7		45. ±16.4	
	No school	31.8 ± 19.7		4.9 ± 14.6		42 ± 19.7		37.9 ± 14.2		42.3 ± 8.1		38.8 ± 10.6	
HCV infection	Positive	28.4 ± 12.4	0.38	45.4 ± 11.9	0.87	39.3 ± 13.5	0.3	39.5 ± 14.3	0.45	41.01 ± 10.7	0.3	38.7 ± 10.6	0.31
	negative	41.4 ± 23.1		51.4 ± 18.9		51.3 ± 23.5		38.1 ± 16.2		47.5 ± 14.7		45.6 ± 16.1	
Pre-transfusion Hb%	< 9 g%	49.9 ± 12.4	0.4	56.2 + 14.7	0.51	41.8 + 11.1	0.28	55.8 + 21.5	0.33	56.7 ± 18.5	0.23	51.2 ± 12.6	0.15
	≥9 g%	50.7 ± 6.6		53.3 + 18.1		45.6 + 12.4		52.9 + 14.8		54.7 ± 5.6		50.9 ± 13.1	
Serum ferritin (ng/ml)	< 2500	41.7 ± 21.6	0.31	48.9 ± 19.1	0.09	52.3 ± 23.5	0.5	36.7 ± 15.1	0.06	46.1 ± 15.9	0.2	44.9 ± 15.1	0.07
	≥2500	32.9 ± 19.7		49.6 ± 15.4		42.8 ± 18.5		40.03 ± 15.9		44.5 ± 12.9		41.9 ± 14.6	
Age of start transfusion	*r*	0.19		0.09		0.2		0.04		0.13		0.17	
	*p*-value	0.1		0.4		0.08		0.7		0.3		0.1	
Age of start chelation	*r*	0.17		0.23		0.09		0.13		0.17		0.19	
	*p*-value	0.1		0.06		0.4		0.2		0.1		0.1	

*HCV* Hepatitis C virus, *Hb* Hemoglobin, *SC* Subcutaneous. * = Significant (*p* < 0.05)

No significant correlations were found between different QoL scores and either the age of starting transfusion or the age of starting chelation (*p* = 0.1 for both).

On the age groups, the children group had significantly higher scores regarding the emotional, social, psychological performance as well as the total HRQoL score (*p* = 0.027, 0.01, 0.01 and 0.009 respectively). Also, the children group had higher but statistically insignificant physical and school performance scores (*p* = 0.9, 0.13 respectively) (Table 3).

**Table 3:** Comparison between different scores regarding the age groups

Parameter	Age 8-12	Age 13-18	*P* value
	*N* = 39	*N* = 25	
Mean Physical score	40.3 ± 2.6	31.7 ± 11.7	0.09
Mean emotional score	53.1 ± 1.8	43.6 ± 6.1	0.027*
Mean Social score	53.4 ± 2.8	39 ± 1.2	0.01*
Mean School performance	40.9 ± 1.6	34.9 ± 3.9	0. 13
Mean Psychological score	47.9 ± 1.5	41.1 ± 4.9	0.01*
Mean Total score	46.8 ± 5.9	37.8 ± 8.7	0.009*

*= Significant (*p* < 0.05)

Regression analysis showed that older age of starting transfusion in thalassemia patients was the most powerful protecting factor from poor physical QoL (OR = 0.96, *p* = 0.03), poor psychological QoL (OR = 0.94, *p* = 0.01) and low total QoL score (OR = 0.94, *p* = 0.01). While hepatomegaly was the most predicting factor for poor physical QoL (OR = 8.5, *p* = 0.02). Household income was the most predicting factor for poor emotional QoL (OR = 5.03, *p* = 0.04). High serum ferritin is the most predicting factor for poor social QoL (OR = 1.1, *p* = 0.04) (Table 4).

**Table 4:** Logistic regression analysis of factors predicting poor different QOL scores

Variable	OR	CI (95%)	*p*- value
*Physical*			
Age of transfusion (Older age)	0.96	0.92-0.96	0.03*
Hepatomegaly	8.5	1.3-52.7	0.02*
*Emotional*			
House hold income (low)	5.03	0.95-26.6	0.043*
*Social*			
Serum ferritin (high)	1.1	1.001-1.1	0.04*
*Psychological*			
Age of transfusion (Older age)	0.94	0.90-0.98	0.01*
*Total*			
Age of transfusion (Older age)	0.94	0.90-0.98	0.01*

OR: Odd Ratio; CI 95%: 95% confidence intervals. * = Significant (*p* < 0.05)

## Discussion

In the present study, assessing the QoL in beta thalassemia major (BTM) children in Minia governorate showed the impact of BTM on the patients’ performance in different aspects of life. The enrolled patients had lower growth parameters, as well as lower physical, social, emotional, school functioning, and total QoL scores compared with their matched healthy peers. Lower growth parameters and QoL scores in BTM patients have recognized observations in previous studies [19, 20] that can be attributed to the chronic anemic state and/or the consequences of iron overload.

Physical performance was the most severely affected parameter in thalassemia patients.

Chronic anemia status with consequent easy fatigability is the most important cause of physical performance deterioration in thalassemia patients. This is aggravated by repeated unavailability of the matched blood and needing to travel long distances from the far rural areas to the central hospital transfusion center. All these factors may lead to irregular blood transfusion and thus aggravate the anemic impact on physical activity, which explains low physical score in thalassemic patients [21, 22].

The low emotional and psychological scores could also be cofactors in the deterioration of physical activities [23–26]. The younger age of starting transfusion was associated with poor HRQoL. A longer period of exposure to the circumstances of repeated transfusion has a great burden on the fitness and physical performance of the thalassemia patients. Moreover, the longer period of exposure to iron metabolites specifically in the liver and other different tissues is an important factor affecting physical performance.

The enrolled BTM patients had low social performance compared to healthy children. The majority of young thalassemia patients can share playing with other kids or being a member of a play team. The absence of evident disease complications at a young age results in decreased feeling of stigmatization. As the patient grows, he recognizes the nature of his disease, he experiences repeated blood transfusion visits and school absence, and the characteristic morphological changes of thalassemia become more evident, all these factors may result in poor self-esteem and expressed negative thoughts about their lives and felt sadness, anger, and hurt toward their chronic illness. Social withdrawal and low social performance will be the result [27].

School functioning subscale scored the lowest in thalassemia patients regardless of the age. Frequent school absence for hospital visits and lack of both mental and physical energies when achieving academic educational activities [20, 28–32].

Chronic anemia, poverty and parents’ illiteracy in most thalassemia patients put schooling and education in the last priority for these patients.

The Psychosocial health summary scores of our patients were lower than healthy controls. Thalassemia patients may suffer from higher levels of depression and anxiety which experience a significant decrease in their psychological wellbeing [33, 34]. This finding seems to support previous studies on psychosocial aspects of thalassemia that more psychosocial support should be given to thalassemia patients [35, 36].

As BTM children get older, the morbidity prevents them from being more productive and a sense of inferiority might develop and lower their emotional domain scores [28]. Also, complications of the disease with its impact on physical morphology, treatment by repeated transfusion and iron chelation therapy with repeated invasive procedure and repeated hospital visits, all are emotionally demanding [27]. Establishment of supportive systems including some psychological consultation schedules in thalassemia clinics are mandatory.

Emotional functioning is one of the impaired domains of thalassemia patients and this study showed that thalassemia patients were lower emotionally functioning. Older children with thalassemia experienced fewer symptoms of depression, reflecting a process of adjustment and coping [37, 38] also suggests that thalassemia patients have their own coping strategies in dealing with their life. These patients seek the ability to satisfy some of their needs on their own and develop a sense of autonomy, but caregiver’s refusal to let them perform these tasks owing to their illness may result in shame and doubt about their ability to handle problems [27]. Poverty can add more complex emotional burden to these patients as the parents cannot provide a comfortable life suitable for the special needs of their children.

In the present study, the type of chelation therapy or the mode of administration had no impact on the different QoL domains. BTM patients have different causes to suffer. Coming from far areas to receive their scheduled transfusion or their planned medications, painful maneuvers either for blood sampling or transfusion, and the early and late complications of thalassemia. All these make the type of iron chelation therapy or the mode of administration to be secondary causes affecting the patient’s QoL.

These results disagree with some previous studies, which reported that the type of iron chelation therapy compromises different aspects of QoL [39–41]. Other authors stated that, in addition to deferoxamine, deferiprone also appears to negatively impact QOL, probably because of its proved side effects [28].

Younger patients can show better QOL compared with older ones. Most of the beta thalassemia complications are cumulative, resulting from the continuous hemolytic process, the consequent of hemosiderosis, and the chance of acquiring transfusion-related infections. In BTM children, as they age, self-awareness increases with more cognition toward their illness status. Their ability to share and take the responsibility is challenged by the disease status. Inferiority sensation might develop which lower their emotional, social and psychological domain scores [27, 28].

### Limitation of the study

This is the first study in Upper Egypt to assess the QOL in thalassemic children. Lack of cooperation of the patients’ caregivers and their denying of many symptoms were the main challenge in this study. Refusal to participate in the study due to traditional thoughts limits the number of patients enrolled in this study.

## Conclusions

In view of our findings, we conclude that quality of life and functioning is affected, thereby showing a need for better care in Egypt. Furthermore, Blood scarcity and transfusion delay may be augmenting the lower quality of life. This necessitates a more integrated planning for health services in Egypt.

## Abbreviations

BTM: B-thalassemia major
HRQoL: Health-related quality of life
QoL: Quality of life
WHO: World health organization
